# Retrodeformation and muscular reconstruction of ornithomimosaurian dinosaur crania

**DOI:** 10.7717/peerj.1093

**Published:** 2015-07-09

**Authors:** Andrew R. Cuff, Emily J. Rayfield

**Affiliations:** School of Earth Sciences, University of Bristol, Bristol, United Kingdom

**Keywords:** Skull, Rhamphotheca, Retrodeformation, Myology, Ornithomimosaurs, Bite forces

## Abstract

Ornithomimosaur dinosaurs evolved lightweight, edentulous skulls that possessed keratinous rhamphothecae. Understanding the anatomy of these taxa allows for a greater understanding of “ostrich-mimic” dinosaurs and character change during theropod dinosaur evolution. However, taphonomic processes during fossilisation often distort fossil remains. Retrodeformation offers a means by which to recover a hypothesis of the original anatomy of the specimen, and 3D scanning technologies present a way to constrain and document the retrodeformation process. Using computed tomography (CT) scan data, specimen specific retrodeformations were performed on three-dimensionally preserved but taphonomically distorted skulls of the deinocheirid *Garudimimus brevipes* Barsbold, 1981 and the ornithomimids *Struthiomimus altus* Lambe, 1902 and *Ornithomimus edmontonicus* Sternberg, 1933. This allowed for a reconstruction of the adductor musculature, which was then mapped onto the crania, from which muscle mechanical advantage and bite forces were calculated pre- and post-retrodeformation. The extent of the rhamphotheca was varied in each taxon to represent morphologies found within modern Aves. Well constrained retrodeformation allows for increased confidence in anatomical and functional analysis of fossil specimens and offers an opportunity to more fully understand the soft tissue anatomy of extinct taxa.

## Introduction

Fossil skulls can offer insights into many aspects of vertebrate ecology and evolution. The cranium hosts the major sensory systems and, along with the mandible and hyolingual apparatus, is responsible for the ingestion of food items. Three-dimensionally preserved skulls provide even greater insight by allowing studies of endocranial morphology (Brochu, 2000; Sanders & Smith, 2005; Witmer & Ridgely, 2009), reconstruction of soft tissues (e.g., rhamphothecae and musculature: Holliday, 2009; Lautenschlager, 2013; Lautenschlager et al., 2013), and functional analysis (Rayfield et al., 2001; Rayfield et al., 2007; Lautenschlager, 2013; Button, Rayfield & Barrett, 2014). However, soft tissue reconstructions in particular are limited by the quality of the specimens on which they are based. This has often posed problems for palaeontologists as taphonomic processes (both pre- and post-burial) can lead to the disarticulation or distortion of skeletal remains. As such, reconstructing and retrodeforming fossil remains can correct for taphonomic damage and is important for furthering our understanding of extinct taxa (Tschopp, Russo & Dzemski, 2013; Williams, 1990).

Various methods have been used to retrodeform fossil taxa. Methods particularly applicable to fossils preserved on a 2D bedding plane range from rescaling drawings (Rushton & Smith, 1993) to the determination of the strain ellipse (Cooper, 1990; Hughes & Jell, 1992) or other ways of deducing tectonic deformation (Motani, 1997). Digital techniques lend themselves to retrodeformation of 3D preserved fossils, including employing 3D computer models for user manipulation of individual disarticulated bones (Lautenschlager, 2013; Porro, Rayfield & Clack, 2015), modifying digital models by reference to closely related extant taxa (Zollikofer et al., 2005; Gunz et al., 2009) or by using landmarks (Molnar et al., 2012; Tallman et al., 2014) and geometric morphometrics (Angielczyk & Sheets, 2007; Hedrick & Dodson, 2013). The efficacies of these methods may be debated, but ultimately they are limited by the quality of preserved material (including brittle and plastic deformation) and perception of what the original specimen should look like, whether informed by symmetry or informed by closely related extant or extinct taxa.

Ornithomimosauria are a clade of coelurosaurian theropod dinosaurs that are commonly known as “ostrich-mimicking” dinosaurs due to their cranial and postcranial convergences with palaeognathous birds. The convergence is seen in their lightweight skulls, with relatively large orbits and edentate jaw margins that bear rhamphotheca (Makovicky, Kobayashi & Currie, 2004). The most primitive members of Ornithomimosauria (*Nqwebasaurus thwazi* De Klerk et al., 2000, and *Pelecanimimus polyodon* Perez-Moreno et al., 1994) possess numerous tiny teeth in the premaxillae, maxillae and mandibles. More derived members of the group lose their upper dentition, maintaining a reduced dentition on the mandible (*Harpymimus okladnikovi* Barsbold & Perle, 1984; and *Shenzhousaurus orientalis* Ji et al., 2003), before becoming fully edentate (as in deinocheirids (Lee et al., 2014) and ornithomimids (Makovicky et al., 2010)). Where teeth are lost, ornithomimids possess beaks, inferred from the presence of foramina on the lateral surfaces the premaxilla, maxilla and mandible and the preservation of remnants of keratinous rhamphothecae in two specimens, the *Ornithomimus* specimen used in this study, RTMP 1995.110.0001, and *Gallimimus bullatus* Osmólska, Roniewicz & Barsbold, 1972, specimen GIN100/1133 (Norell, Makovicky & Currie, 2001). The posterior extent of the beak is subject to debate, yet important for functional considerations as it provides a food capture and manipulation surface and plays a role in the reduction of feeding-related bony stress (Lautenschlager et al., 2013).

In addition to the rhamphotheca, variation in other soft tissues has important functional consequences for the skull. Many studies have attempted to reconstruct the adductor musculature anatomy of a wide range of taxa across the Dinosauria: ankylosaurs (Haas, 1969); hadrosaurs (Bell, Snively & Shychoski, 2009; Holliday, 2009); Marginocephalia (Haas, 1955; Holliday, 2009; Sereno, Zhao & Tan, 2010); prosauropods (Fairman, 1999); sauropods (Haas, 1969; Holliday, 2009; Young et al., 2012) and theropods (Adams, 1919; Rayfield et al., 2001; Holliday, 2009; Bates & Falkingham, 2012; Lautenschlager, 2013). The studies range from simple identification and line drawings based on osteological correlates (e.g., Haas, 1969), to clay modelling of the muscles (Rayfield et al., 2001), to digital reconstructions (e.g., Lautenschlager, 2013). The increased sophistication of adductor reconstruction has permitted more accurate estimation of not just the size of individual muscles, and therefore the force they can potentially generate, but their spatial relations to each other and effects of muscle bulging during contractions.

The aim of this paper is to document the process and consequences of retrodeformation of the crania of three ornithomimosaur theropod dinosaurs. Then using our hypotheses of retrodeformed morphology we reconstruct the comparative adductor muscle anatomy and calculate and compare the relative differences between adductor mechanical advantage and the resulting estimated bite force along the jaw. We do this for skulls pre- and post-retrodeformation, to deduce, in the context of the specimens presented here, the influence of retrodeformation on our predictions of function. This allows characterisation of bite forces arising during the evolution of edentulism between the ornithomimids and deinocheirids and more broadly within the ornithomimosaurs, one of at least three clades of coelurosaurian theropods that diverge from hypercarnivory (Zanno & Makovicky, 2011). We compare our predicted bite forces to the only other estimate from a herbivorous theropod, *Erlikosaurus andrewsii* Perle, 1981, a therizinosaur (Lautenschlager et al., 2013). Given that the three ornithomimosaurians and *E. andrewsii* have similar sized skulls, we test for congruence in bite force magnitudes between these putatively herbivorous taxa.

## Methods

### Specimens

Few well preserved, three-dimensional ornithomimosaur skulls are known. Here we focus on crania from three taxa: *Garudimimus brevipes*, *Struthiomimus altus* and *Ornithomimus edmontonicus*. *Garudimimus* is known from only a single specimen. Our chosen specimens of *S. altus* and *O. edmontonicus* represent the best prepared material for either taxon. There are other cranial remains, but most are badly crushed, encased within matrix prohibiting detailed observation, or remain taxonomically contentious. A number of specimens were examined first hand (see Appendix S1) and information from the published literature on the well preserved skulls of *Gallimimus* (Osmólska, Roniewicz & Barsbold, 1972), *Deinocheirus* (Lee et al., 2014), and *Sinornithomimus* (Kobayashi & Lü, 2003), was used for comparison where possible and inform on the retrodeformation process.

The specimen of *Garudimimus brevipes* (GIN 100/13, described by Barsbold (1981) and Kobayashi & Barsbold (2005)) was scanned at the University of Texas using a P250D scanner at 419 kV, 1.8 mA, aluminum filter, slice thickness = 0.5 mm, total slices = 517. The *Ornithomimus edmontonicus* specimen (RTMP 1995.110.0001) was scanned along the coronal axis for a total of 420 slices (0.63 mm thickness) with a GE LightSpeed Plus CT scanner (Tahara & Larsson, 2011). The *Struthiomimus altus* specimen (RTMP 1990.026.0001) was scanned using the same parameters as the *Ornithomimus* specimen, creating a dataset of 416 slices along the coronal axis. For both *Ornithomimus* and *Struthiomimus* the scans are of relatively low quality. To provide better detail, the scans were upsampled in *Avizo* 7.0 (FEI Visualization Sciences Group, USA). This process creates interpolations between each of the original CT slices to provide twice the number of slices in every axis for smoother reconstructions, but not providing any further resolution. The *Garudimimus* CT dataset was not resampled.

### Reconstructions

The CT datasets were loaded into the visualisation and analysis package *Avizo 7.0*. Segmentation and isolation of each individual cranial bone was performed, as far as the deformed, and in some places incomplete, datasets permitted. As all of the specimens suffered deformation, it was necessary to undertake retrodeformation to provide a complete undeformed skull for each species on which the soft tissue reconstructions could be based. Notably, the nature and magnitude of deformation differed in each taxon, and hence specimen-specific retrodeformation processes were applied to each specimen. Furthermore, there is no known undistorted skull for any of the taxa studied. The process of deformation was therefore informed by the topographic relationships of the individual cranial elements in the 3D dataset, evidence of breakage and cracks revealed from direct observation of specimens and the CT scan data, and information gathered from related ornithomimosaur material from museum collections and the literature (as outlined above, and see Appendix S1). Where possible, a set of criteria were employed to perform and constrain the process. As outlined in Arbour & Currie (2012), the shape of the orbit was used a proxy to determine the degree of deformation. Orbital retrodeformation was therefore employed to reconstruct the arrangement of the surrounding facial bones. In all studied ornithomimosaurs, both actual specimens and literature study, the pattern of breakage and deformation to the bones of the orbital region suggest that the orbits in undeformed taxa should be approximately circular. As such, this was the first correction applied to the *Garudimimus* and *Struthiomimus* skulls. In *Garudimimus*, the individual bones were segmented from the CT scan datasets and the bones surrounding the orbit were rotated into position using the editing tools in *Avizo* (sensu Lautenschlager et al., 2013; Button, Rayfield & Barrett, 2014). This process was sequentially repeated with bones further from the orbits, until all of the right side of the specimen was reconstructed with the original material (no obvious plastic deformation was seen in this specimen except for the posterior margin of the maxilla). In the *Struthiomimus* skull, the orbital region was dorsally shifted by translating the bones within the “edit label field” function in *Avizo* until a circular orbit was restored. This process was continued anteriorly and posteriorly until a smooth cranial roof was created. The orbit was then measured in anteroposterior and dorsoventral axes with the “measure” tool within *Avizo* to check whether a near circular structure has been achieved via the retrodeformation process.

In ankylosaur skulls, it was noted that the bones of the palate suffered little deformation (Arbour & Currie, 2012). This was also true for the specimens studied here, although the palatines and pterygoids in *Ornithomimus* were mediolaterally displaced and overlapped. As such, palatal morphology and width were used as a marker to determine the mediolateral dimensions and required expansion of the skulls. For *Ornithomimus* the palatal bones were separated and aligned, and the remainder of the skull expanded mediolaterally to fit the palate. The palatal morphology of observed and well preserved specimens in the literature was used to inform on this procedure.

For the remaining bones it was possible to determine whether cortical bone had collapsed or was damaged using the CT scan data, so that the surface topography of the bones could be reconstructed using the paintbrush region-selecting tool within *Avizo* to match that seen in other specimens or ornithomimosaur taxa (e.g., the jugals in *Ornithomimus*). In some places bone was so badly damaged that full reconstruction required material from the other scans and digital manipulation using the paintbrush tools to create “new bone”. This was always informed by the individuals studied here as well as other specimens and taxa from museum collections and the literature. For example, in the anterior portion of the jugal in *Garudimimus*, the bone is broken and partially missing, but should overlap the posterior ramus of the maxilla and contact the lacrimal. The maxillary ramus was therefore ventrally displaced to bring it into alignment with the preserved remains of the jugal (as described for the orbit of *Struthiomimus*), and the jugal was extended using the paintbrush tool in three dimensions to provide the required contact whilst maintaining the shape seen in the other scanned and observed ornithomimosaurs. In *Garudimimus* the right side of the skull was better preserved than the left, whilst the opposite was true in *Struthiomimus*. Bones of the better preserved sides, once aligned and reconstructed, were mirrored about the sagittal midline of the skull, using the mirror function in *Avizo* (Lautenschlager, 2013).

### Ornithomimosaur myology

Following methods of Holliday (2009), Lautenschlager (2013) and Button, Rayfield & Barrett (2014), the individual insertions and origination sites for the adductor muscles were digitally mapped onto the 3D ornithomimosaur skull reconstructions. Where there was a lack of osteological correlates on the bones in either the CT scans or the actual specimens, phylogenetic bracketing was used to ascertain likely insertion and origination locations. These originations and insertions were demarcated on the skull and mandible. As there was no scanned *Garudimimus* jaw, the *Struthiomimus* jaw was used (scaled and rotated into place) to ascertain muscle orientation as it was the closest in morphology of the two ornithomimids.

For each of the individual muscles, a number of simple rods were used to connect the limits of the origins and insertions (following Curtis et al., 2008). This process was used to assess the margins of the muscles and ensure there was no overlap with either the bone or other muscle bodies. In places, these rods were manually wrapped around the bones within *Avizo*. In other reconstructions, the neurovascular system also has been used (Lautenschlager, 2013), but its canals were not readily traceable *in-silico* from our lower quality CT scans. In museum specimens with matrix-obscured neurocrania, these canals were not visible either. Muscles were fully ‘fleshed’ by connecting all of the rods belonging to the same muscles until they were all merged to form a single “muscle” (Lautenschlager, 2013; Button, Rayfield & Barrett, 2014). This process was repeated for all adductor muscles. All of the fleshed out muscles were then enlarged until they occupied the maximum amount of space within the chambers without intersecting in three-dimensional space, which *Avizo* can prevent. The expanded muscle bodies were then digitally smoothed using tools in *Avizo*.

Muscle forces were estimated using the dry skull method (Thomason, 1991), where force (*F*_mus_) equals the cross-sectional area (CSA) multiplied by the isometric muscle stress (*σ* here taken as 0.3 N mm^−2^: Weijs & Hillen, 1985; Thomason, 1991):

\begin{eqnarray*} {F}_{\mathrm{mus}}=\mathrm{CSA}\times \sigma . \end{eqnarray*}

The CSA is calculated in *Avizo*, using the ‘clipping plane’ tool to define the cross section and the ‘material statistics’ module which calculates the surface area. This was done for each muscle at its widest location to give the maximum CSA and thus maximum estimated force. As this method fails to take into account pennation angle of muscle fibres, the forces were multiplied by a scale factor (calculated from experimental comparisons between modelled and actual data (Thomason, 1991)) of 1.5 to compensate. Given the arrangement of muscle bodies, the total muscle force is the resultant of anteroposterior, dorsoventral and mediolateral force components. Mediolaterally orientated muscle force has limited influence on jaw closing due to the almost vertical orientation of the muscle lines of action. As such the dorsoventral component is studied for bite force lever mechanics (as in Lautenschlager, 2013). The force of each muscle (*F*_mus_: Table 2) can be multiplied by the perpendicular distance of the muscle centroid from the jaw joint (measured in Avizo) to provide a muscle moment:

\begin{eqnarray*} {F}_{\mathrm{in}}={F}_{\mathrm{mus}}\times \text{perpendicular distance from joint}. \end{eqnarray*}

The sum of each of the muscle input moments can then be used to calculate bite forces (*F*_bf_) at individual locations along the skull (Table 2):

\begin{eqnarray*} \text{Total}~{F}_{\mathrm{in}}=\Sigma ({F}_{\mathrm{mus}}\times \text{perpendicular distance from joint}) \end{eqnarray*}

\begin{eqnarray*} \mathrm{Total}~{F}_{\mathrm{in}}=\mathrm{Total}~{F}_{\mathrm{out}} \end{eqnarray*}

\begin{eqnarray*} \mathrm{Total}~{F}_{\mathrm{out}}={F}_{\mathrm{bf}}\times \text{perpendicular distance from joint}. \end{eqnarray*}

### Rhamphothecae

**Figure 1:**
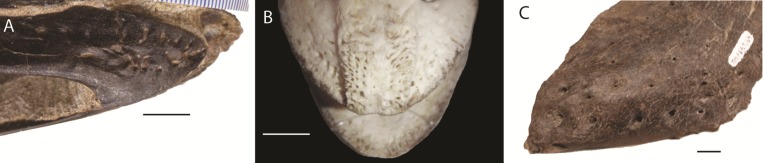
Foramina and rugosities in the rostra of certain taxa. (A) Anterior, right mandible of *Struthiomimus altus* (RTMP 1990.026.0001); (B) Dorsal view of anterior premaxilla of ostrich and mandible (ROM R1080); (C) Anterior dentary of a tyrannosaur (*Daspletosaurus*?) RTMP (1967.009.0164). Scale bars = 1 cm.

Foramina are regularly cited as evidence for a keratinous rhamphotheca (e.g., Kobayashi & Lü, 2003). In modern birds, foramina can be found on the surface of the anterior premaxilla and mandible, where the rhamphothecae may be expected to be thickest (Fig. 1) (see Morhardt, 2009). In extant palaeognaths, the beak provides a close sheath over the bones of the mandible and skull (Davies, 2003), whereas in neognaths the rhamphotheca extends well beyond the oral margins. In many species, the beak also extends well beyond the anterior margins of the bone; in extreme examples such as hornbills and toucans, the rhamphotheca may be two to three times longer than the amount of bone it covers (Seki, Bodde & Meyers, 2010).

In non-avian theropods, the picture is more complicated. Ornithomimosaurs, oviraptorids, therizinosaurs, and *Limusaurus* (a ceratosaur) underwent tooth loss leading to partial edentulism and inferences of rhamphothecae (Zanno et al., 2009; Zanno & Makovicky, 2011). These taxa bear regular foramina across the lateral surface of edentulous regions of the premaxilla and dentary. There are also grooves on the mandible of *Erlikosaurus* (a therizinosaur) that appear to demarcate a keratinous rhamphotheca/beak (Lautenschlager, 2013; Lautenschlager et al., 2013). However, neurovascular foramina are also present in large theropods (e.g., tyrannosaurs: Fig. 1; spinosaurs: Dal Sasso et al., 2005; Morhardt, 2009) where teeth are present and keratinous beaks are not inferred.

As the presence of foramina is not a reliable characteristic for modelling rhamphothecae, we must rely on other lines of evidence. Because ornithomimosaurs (and other edentulous theropods) had downturned dentaries, the jaws do not occlude across the entire oral margin (Zanno et al., 2009; Zanno & Makovicky, 2011). As this would limit the functionality of the jaws, it is reasonable to expect the rhamphotheca to fill the gap to form an occlusal surface. Preserved rhamphothecae also exist on two ornithomimid specimens. In *Ornithomimus* RTMP 1995.110.0001 (the specimen used in this analysis) the rhamphotheca is around 4.30 mm in dorsoventral depth on both the upper and lower jaws. This is similar to a remnant of rhamphotheca approximately 3.0 mm depth on the *Gallimimus* specimen (GIN 100/1133) (measured from Norell, Makovicky & Currie, 2001). Assuming that the jaws occluded along their oral margins, the rhamphotheca was modelled here in all taxa to fill the oral margins, deeper at the anterior (using the preserved specimens as indicating a minimum dorsoventral thickness) and tapering posteriorly (as in modern birds). Two reconstructions accommodated uncertainty about the extent of the rhamphotheca beyond the oral margins, and two morphologies were made for the skull. These include: a conservative, ‘small’ beak model that is modelled on an ostrich beak, with limited extension of the rhamphotheca around the nares; and a more extensive ‘big’ beak model where the beak margins border the antorbital fossa. In neornithines, a naricorn rhamphothecal plate covers variable extents of the nares depending on the species (Hieronymus & Witmer, 2010), and we have taken a conservative approach by not covering any of the nares. In addition, we have not covered any of the antorbital fossa similar to the practice of Lautenschlager et al. (2013) (Fig. 7), who did however partially cover the larger nares of *Erlikosaurus* (Lautenschlager et al., 2013). As the lower jaw was not used in any functional studies, beaks were not reconstructed for the mandibles.

## Results

**Figure 2:**
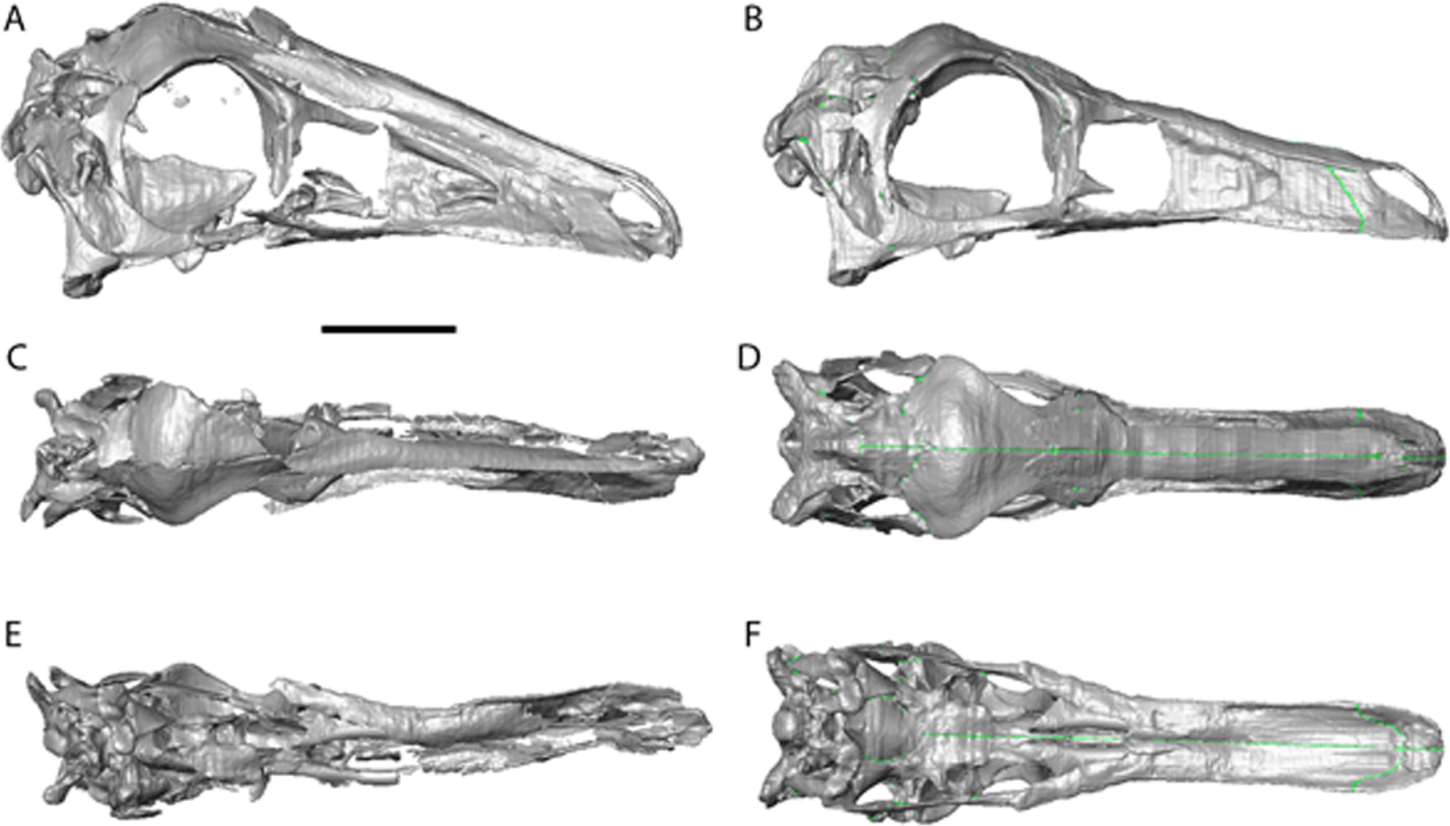
*Garudimimus brevipes* reconstruction (GIN 100/13). (A), (C), (E), original skull, (B), (D), (F), retrodeformed skulls. (A), (B), right lateral; (C), (D) dorsal; (E), (F), ventral views. Scale bar = 5 cm. See Video S1 and Video S2 showing video of the skull before and after retrodeformation.

**Figure 3:**
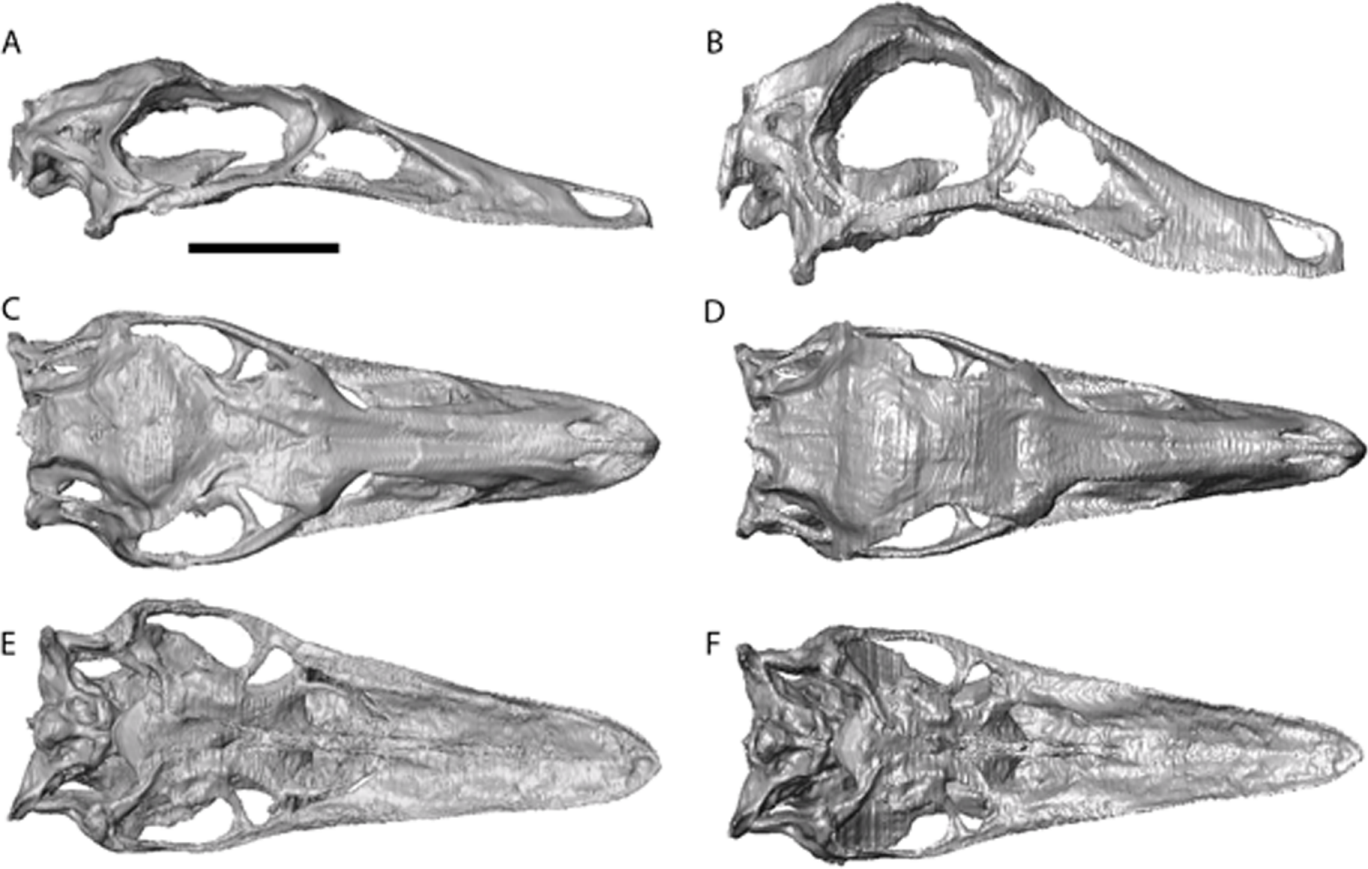
*Struthiomimus altus* reconstruction (RTMP 1990.026.0001). Note the dorsoventral expansion of the skull after retrodeformation, particularly of the orbital region. (A), (C), (E), original skull, (B), (D), (F), retrodeformed skulls. (A),(B), right lateral; (C), (D) dorsal; (E), (F), ventral views. Scale bar = 5 cm. See Videos S3 and S4 showing video of the skull before and after retrodeformation.

**Figure 4:**
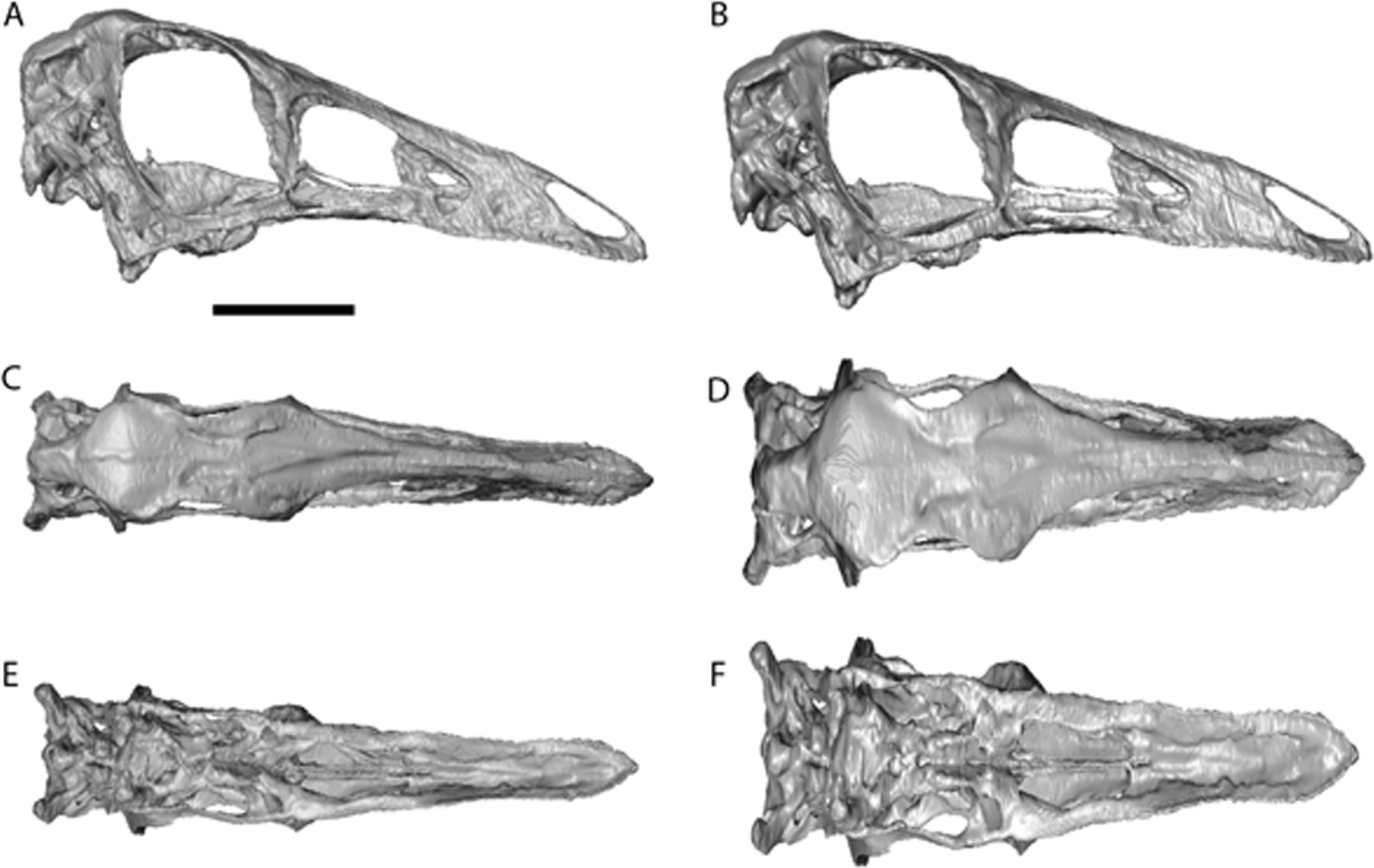
*Ornithomimus edmontonicus* reconstruction (RTMP 1995.110.0001) showing the effect of the mediolateral expansion after separating the taphonomically deformed bones of the palate. (A), (C), (E), original skull, (B), (D), (F), retrodeformed skulls. (A), (B), right lateral; (C), (D) dorsal; (E), (F), ventral views. Scale bar = 5 cm. See Videos S5 and S6 showing video of the skull before and after retrodeformation.

**Figure 5:**
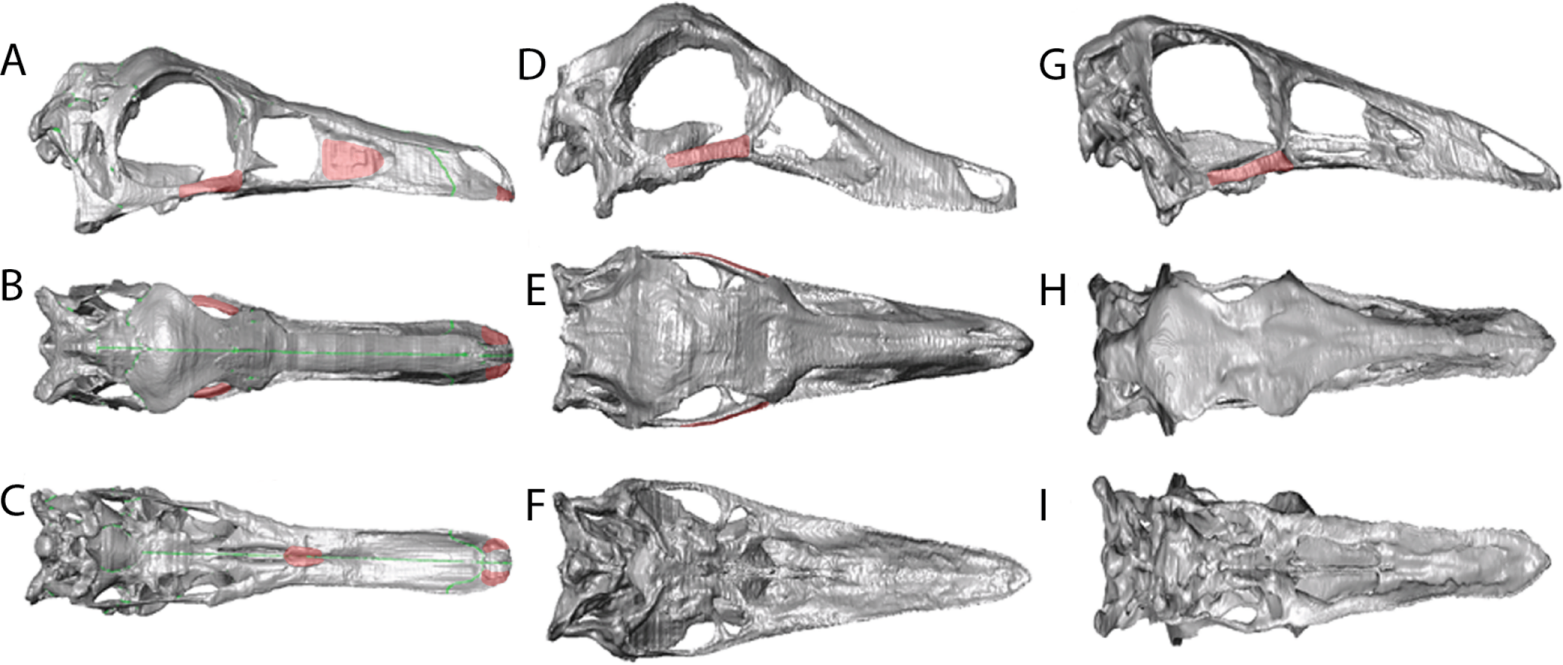
Reconstructions showing the regions where material was added using the *paintbrush* region-selecting tool within *Avizo.* Regions in red showing the areas where new material was added. (A)–(C) *Garudimimus*, (D)–(F) *Struthiomimus*, (G)–(I) *Ornithomimus*.

The cranial reconstructions are shown in Figs. 2–4. No new gross anatomical descriptive information is revealed but the overall dimensions of the skull are modified by retrodeformation (Table 1). The width of the skull is modified in all taxa post-retrodeformation, as are the dimensions of the orbit in *Garudimimus* and *Struthiomimus*. The few areas where cranial material was digitally added compared to original bone can be seen in Fig. 5.

**Table 1:** Selection of measurements pre- and post-retrodeformation for each skull. Length is measured from the centre of the quadrate condyle to the tip of premaxilla; width is measured as the distance between the centres of each quadrate condyle; orbit height is measured as the dorsoventral height of the centre of the orbit. All measures are in millimetres.

	*Garudimimus*		*Struthiomimus*		*Ornithomimus*	
	Pre-	Post-	Pre-	Post-	Pre-	Post-
Length	226	225	183	183	185	185
Width	34^a^	46	64^a^	56	26	42
Orbit height	59.5	61	35	54	68	68

**Notes.**

a Where there is an anterior–posterior offset resulting in a shear, inflating the measure.

**Table 2:** Reconstructed muscle originations and insertions for the ornithomimosaurs studied here (see text for muscle abbreviations).

Muscle	Origination	Insertion
AMEM	Posterior portion of supratemporal fossa	Posterior, mediodorsal edge of mandible
AMEP	Medial portion of supratemporal fossa	Mandibular margin anterior to AMEM insertion
AMES	Medial edge of supratemporal bar	Dorsolateral edge of mandible
AMP	Lateral surface of quadrate	Posterior medial margin of mandibular fossa
PSTs	Rostromedial portion of temporal fossa	Rostromedial mandibular fossa
PTd	Dorsal surface of rostral portion of pterygoid and palatine	Medial surface of articular
PTv	Caudoventral surface of pterygoid	Lateral surface of articular and angular

The *Garudimimus* specimen is the most damaged skull with a fragmentary left side, and fairly complete, but disarticulated, right side cranial elements (Kobayashi & Barsbold, 2005; Fig. 2). Here the right side elements were digitally realigned. The anterior process of the jugal is broken, as is the posterior ramus of the maxilla. The posterior ramus of maxilla was aligned so that the buccal margins of the maxilla formed a continuous, approximately linear, margin. The jugal was reconstructed anteriorly so that it overlapped the maxilla and contacted the lacrimal. When the right side was fully reconstructed, it was mirrored about the sagittal plane to create a complete skull. The palate remained incomplete after mirroring, with the vomers poorly preserved (only a possible fragment exists). The vomers were reconstructed based on the shape and size of those found in the *Struthiomimus* specimen (Figs. 2E and 2F) as this is one of the better preserved and prepared skulls available to study.

The dorsoventral compression in *Struthiomimus* was removed by dorsoventrally expanding the regions dorsal and posterior to the orbit until the orbit was approximately circular (as seen in other ornithomimids (Makovicky, Kobayashi & Currie, 2004)) (Fig. 3). There is also a slight asymmetrical mediolateral shearing, particularly of the left side, so the right side of the skull was mirrored to create exactly the same bones for the left side. Only after CT scanning was it possible to make a more accurate estimate of the extent of the mediolateral crushing in *Ornithomimus*. Using the palate, which is obscured by matrix on the actual specimen, it is possible to see that the elements from each side of the palate have overlapped rather than flattened (Figs. 4E and 4F). By separating the palatal elements using *Avizo* 7.0 and realigning to life position, the width of the palate was recreated. The skull was then expanded so that the palate would fit between the medial surfaces of the facial bones (Fig. 4). In addition to this, the anterior processes of the jugals are crushed on both sides. This likely occurred when the thin cortical bone in the region collapsed into the medial trabecular bone regions, and as such the jugals were reconstructed in these areas (Figs. 4A, 4B and 5).

### Myology

**Figure 6:**
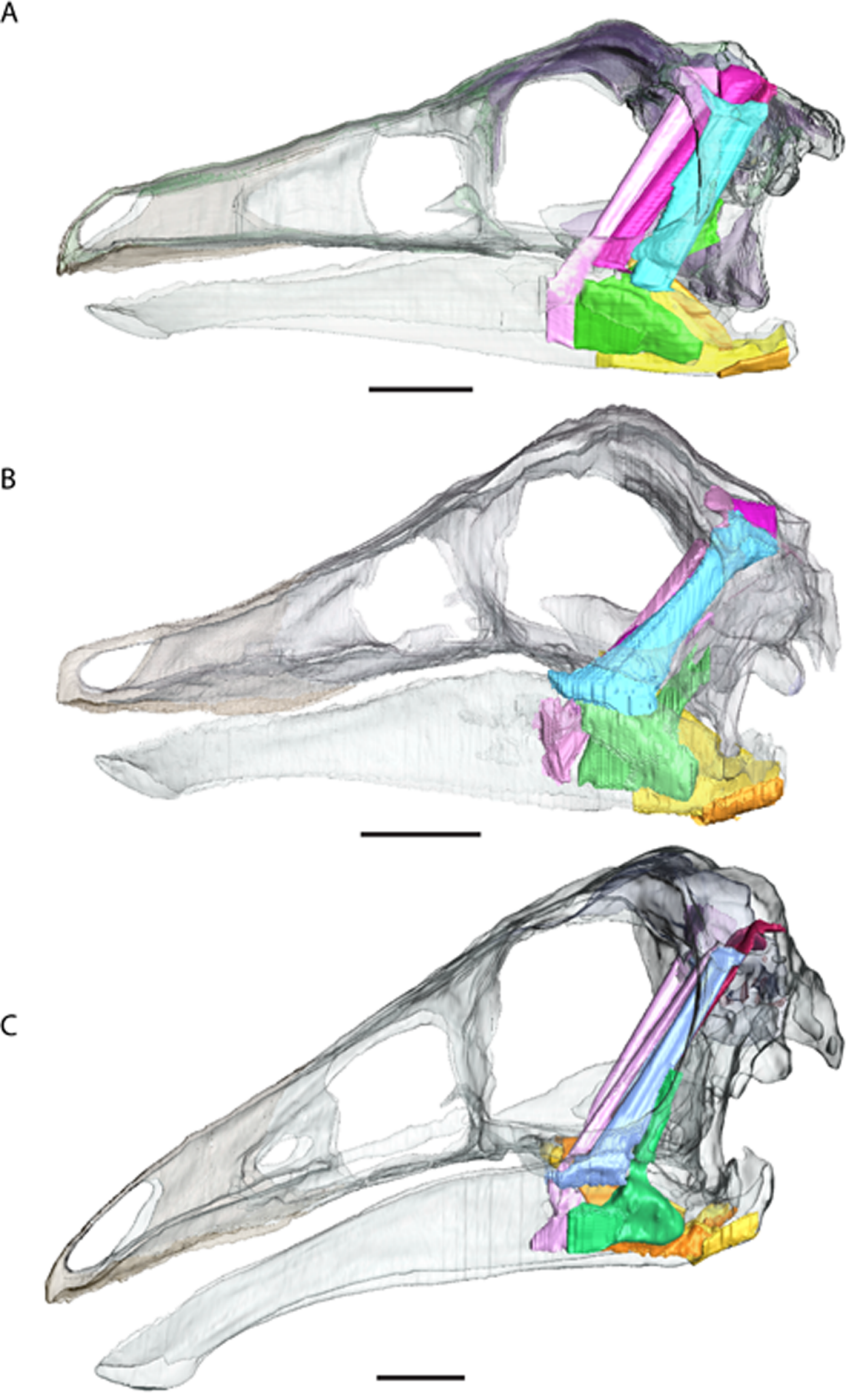
Full cranial reconstruction including musculature of the jaw. (A) *Garudimimus*, (B) *Struthiomimus*, (C) *Ornithomimus.* Scale bars = 5 cm. Pink, PSTs; purple, AMEp; red, AMEm; blue, AMEs; green, AMP; yellow, PTd; orange, PTv.

The reconstructions do not find any major differences between insertions and originations of the ornithomimosaurian myology and other dinosaurs (Fig. 6 and Table 2), except that we could not reliably restore the M. pseudotemporalis profundus. This muscle usually attaches on the epipterygoid in extant sauropsids and has been identified in other dinosaurs (Holliday, 2009). Because none of the specimens had an identifiable epipterygoid attachment visible on the quadrate (as in birds: Holliday & Witmer, 2007) the muscle was not reconstructed. It is possible the muscle occupies some of the space used here in the reconstruction of the M. adductor mandibulae posterior.

**Figure 7:**
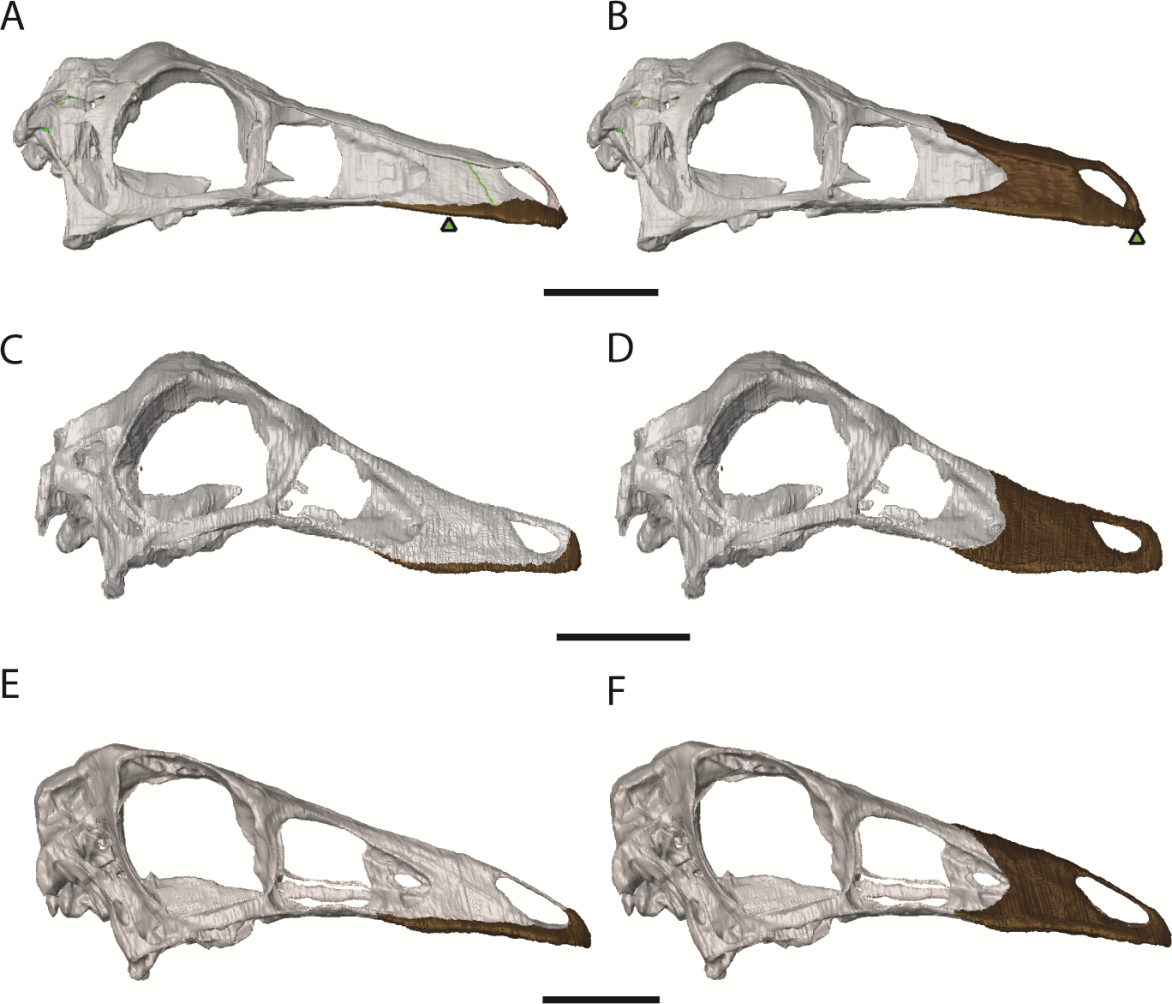
Ornithomimosaur beaks. (A) Small and (B) big beak morphs on *Garudimimus*; (C) small and (D) big beak morphs on *Ornithomimus*; (E) small and (F) big beak morphs on *Struthiomimus*. Scale bars = 5 cm. Triangles represent bite locations for mid-beak and tip of the beak bites (Table 6).

**Figure 8:**
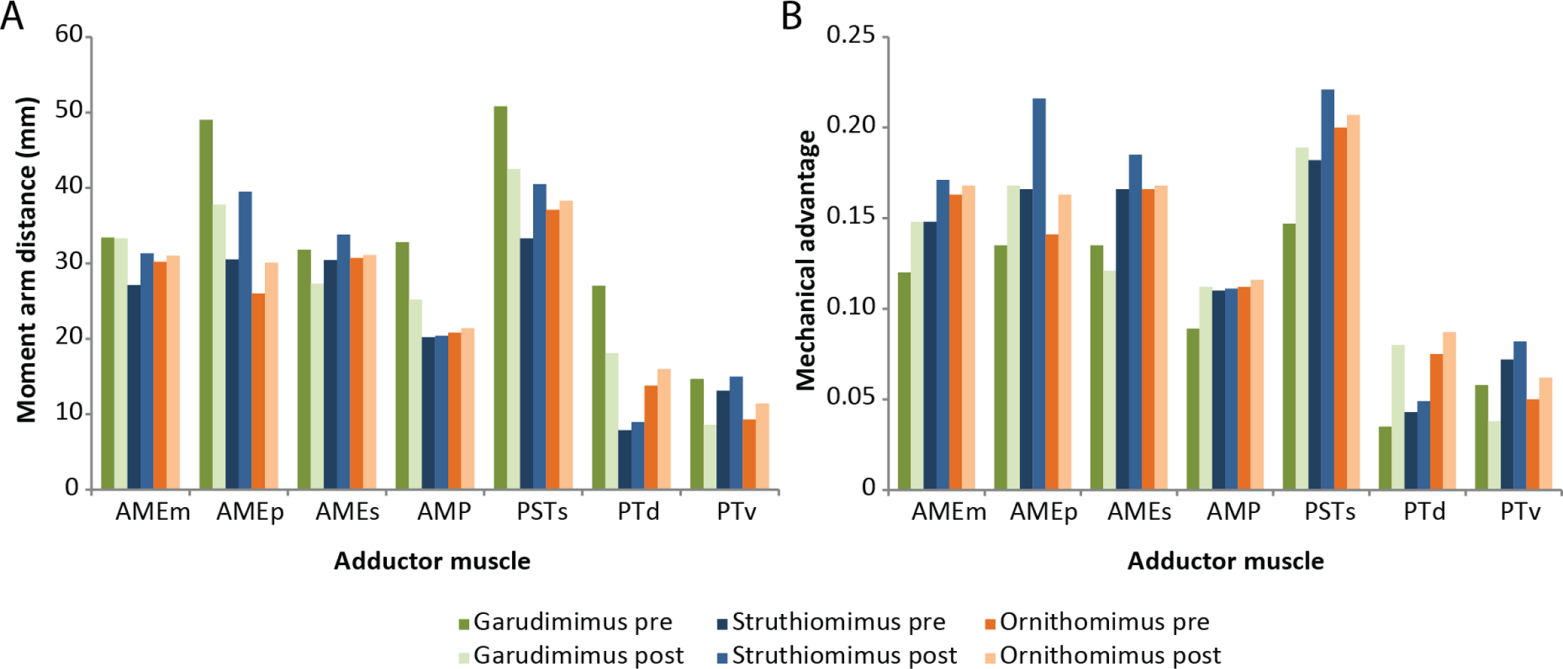
Effects of retrodeformation on myological reconstructions. (A) Moment arm distances, (B) Mechanical advantages. ‘Pre’ and ‘Post’ refer to pre- and post-retrodeformation.

The amounts by which muscle moment arm lengths and mechanical advantages are affected by retrodeformation are variable between taxa and between different muscle groups (Tables 3–5 and Fig. 8). Muscle moment arms and mechanical advantages are modified most in *Garudimimus* and least in *Ornithomimus*. The M.AMEm, M.AMEs and the M.AMP are least affected by retrodeformation. The M.AMEp and the pterygoideus complex are most affected by retrodeformation. Comparison between species shows that for all three ornithomimosaurs the mechanical advantage for the pterygoideus complex is always very low pre- and post-retrodeformation, because the muscle centroids are close to the jaw joint (Table 4). The rest of the muscles possess broadly similar mechanical advantages (Table 4). Using the muscle moment arms and PCSA estimates, muscle forces were calculated (Table 6). There are some notable differences in comparable adductor muscle forces. For example, *Ornithomimus* has typically less forceful muscle contraction, with the exception of the M. pterygoideus dorsalis. *Struthiomimus* and *Garudimimus* have broadly comparable adductor muscle force production with the exception of lower force production in the M. pterygoideus complex of *Garudimimus*. *Struthiomimus* produces the highest total adductor force. Given that the skulls are all similar lengths and therefore the ‘out’ lever arms (jaw lengths) are similar in length, *Struthiomimus* produces the highest bite forces at any of the positions along the jaw, whilst *Ornithomimus* produces the lowest. The presence of a rhamphotheca marginally reduces estimated bite forces.

**Table 3:** Muscle moment arms and mechanical advantages for the specimens prior to retrodeformation. The mechanical advantage out-lever was calculated as the distance from the jaw joint to the anterior tip of the premaxilla with no rhamphothecae: *Garudimimus* = 226 mm; *Struthiomimus* = 183 mm; *Ornithomimus* = 185 mm.

	Moment arm distances (mm)			Mechanical advantage (jaw tip-joint)		
	*Garudi.*	*Struthio.*	*Ornitho.*	*Garudi.*	*Struthio.*	*Ornitho.*
AMEm	33.4	27.1	30.2	0.120	0.148	0.163
AMEp	49.0	30.5	26.0	0.135	0.166	0.141
AMEs	31.8	30.4	30.7	0.135	0.166	0.166
AMP	32.8	20.2	20.8	0.089	0.110	0.112
PSTs	50.8	33.3	37.1	0.147	0.182	0.200
PTd	27.0	7.90	13.8	0.035	0.043	0.075
PTv	14.7	13.1	9.3	0.058	0.072	0.050

**Table 4:** Muscle moment arms and mechanical advantages for the specimens after retrodeformation. The mechanical advantage out-lever was calculated as the distance from the jaw joint to the anterior tip of the premaxilla with no rhamphothecae: *Garudimimus* = 225 mm; *Struthiomimus* = 183 mm; *Ornithomimus* = 185 mm.

	Moment arm distances (mm)			Mechanical advantage (jaw tip-joint)		
	*Garudi.*	*Struthio.*	*Ornitho.*	*Garudi.*	*Struthio.*	*Ornitho.*
AMEm	33.3	31.3	31.0	0.148	0.171	0.168
AMEp	37.8	39.5	30.1	0.168	0.216	0.163
AMEs	27.3	33.8	31.1	0.121	0.185	0.168
AMP	25.2	20.4	21.4	0.112	0.111	0.116
PSTs	42.5	40.5	38.3	0.189	0.221	0.207
PTd	18.1	8.95	16.0	0.080	0.049	0.087
PTv	8.6	15.0	11.4	0.038	0.082	0.062

**Table 5:** Percentage change in muscle moment arms and mechanical advantage after retrodeformation.

	Moment arm distances (mm)				Mechanical advantage (jaw tip-joint)			
	*Garudi.*	*Struthio.*	*Ornitho.*	*Muscle group sum^a^*	*Garudi.*	*Struthio.*	*Ornitho.*	*Muscle group sum^a^*
AMEm	−0.3	15.5	2.6	**18.4**	23.3	15.5	3.1	**41.9**
AMEp	−22.9	29.5	15.8	**68.2**	24.4	30.1	15.6	**70.1**
AMEs	−14.2	11.2	1.3	**26.7**	−10.4	11.4	1.2	**23.0**
AMP	−23.2	1.0	2.9	**27.1**	25.8	0.9	3.6	**30.3**
PSTs	−16.3	21.6	3.2	**41.1**	28.6	21.4	3.5	**53.5**
PTd	−33.0	13.3	15.9	**62.2**	128.6	14.0	16.0	**158.6**
PTv	−41.5	14.5	22.7	**78.7**	−34.5	13.9	24.0	**72.4**
Sum of % change	**−151.4**	**106.6**	**64.4**		**275.6^a^**	**107.2**	**67.0**	

**Notes.**

a Sum of absolute percentage change.

**Table 6:** Muscle loads and bite forces as calculated from muscle reconstructions for each ornithomimosaur. All forces in Newtons. Positions for mid beak (half the distance from the rostral to distal margins of the rhamphothecae) and tip of beak bites are shown in Fig. 7.

	*Garudimimus*	*Ornithomimus*	*Struthiomimus*
AMEm	14.1	8.69	24.1
AMEp	29.0	12.9	28.3
AMEs	17.2	10.5	31.7
AMP	14.3	15.0	13.2
PSTs	23.7	10.4	30.7
PTd	3.17	17.1	40.4
PTv	8.56	7.08	35.3
Tip of beak	19.0	22.0	57.6
Mid beak	23.9	28.6	75.2

## Discussion

Retrodeformation has previously been used to gain a better understanding of the musculoskeletal anatomy of skulls (e.g., Lautenschlager, 2013), which was largely limited to well preserved specimens (Rayfield et al., 2001; Holliday, 2009). The reconstructions here were based on specimen specific taphonomic distortion and relied on knowledge of other well preserved ornithomimosaurs. By restoring the skulls to our interpretation of their original shapes, improved confidence in muscle anatomy and muscle and bite force calculation is now possible. The retrodeformation process influenced measurements of muscle moment arms and calculation of mechanical advantage by variable degrees depending on the amount of deformation in the original specimen. The *Garudimimus* specimen is mediolaterally compressed and dorsoventrally sheared and the snout is bent along its long axis. Correcting for these deformations lead to notable differences between the myological reconstructions before and after retrodeformation (Table 5 and Fig. 8). Widening the *Ornithomimus* skull and making the *Struthiomimus* skull taller and narrower influenced functional variables, but to a lesser degree. This demonstrates the importance of performing retrodeformations to fully understand ornithomimosaur biomechanics. The degree to which functional performance metrics such as bite force and skull stress are influenced by changing skull proportions are also dependent on the relative sizes of muscle groups and therefore the force each group can generate, but our study highlights the importance of retrodeformation in general. Ornithomimosaurs appear to generate relatively low bite forces (Table 6), particularly when considering the body size of the taxa studied here (97.8–195 kg) (Zanno & Makovicky, 2013). The only major difference in muscular performance between the deinocheird *Garudimimus* and the two ornithomimids is that most muscles are more mechanically advantageous within *Ornithomimus* and *Struthiomimus*. This is mainly linked to the longer skull in *Garudimimus*. *Garudimimus* has the smallest bite force, although this calculation may be limited by having to use the mandible of *Struthiomimus* for the *Garudimimus* reconstruction or that the *Garudimimus* specimen used has been described as sub-adult (Kobayashi, 2004). Most known ornithomimosaurs with preserved skulls are relatively small (Zanno & Makovicky, 2013), but the recently described skull of *Deinocheirus mirificus* is 1.02 m in length (Lee et al., 2014). This large, derived (almost hadrosaurid-like) skull has relatively small temporal fenestrae so may have had small adductor muscles (Lee et al., 2014). This, combined with the long rostrum, suggests it too had a relatively small bite force despite its large size. This likely has a consequence on its diet: *Deinocheirus* is known to have consumed small fish based on stomach contents, but is also believed to have consumed plant matter, as hypothesized for other ornithomimosaurs.

Ornithomimosaur bite forces are the lowest reported to date for any non-avian theropod and are lower than those found in another putatively herbivorous theropod (Zanno et al., 2009; Zanno & Makovicky, 2011), *Erlikosaurus* (Lautenschlager, 2013). In that study, it was suggested that such low bite forces (43–134 N depending on location of the bite along the jaw) combined with a keratinous rhamphotheca, could be used to help hold plant material, whilst neck musculature (Rayfield, 2004; Snively & Russell, 2007) provided a ventrocaudal force to strip vegetation (Lautenschlager, 2013; Lautenschlager et al., 2013; Button, Rayfield & Barrett, 2014). This may be a valid method of food acquisition in ornithomimosaurs but further study is required. There are few estimates of bite force in other herbivorous dinosaur taxa. For Sauropoda, estimates of between 235–324 N and 982–1859 N have been calculated for *Diplodocus* and *Camarasaurus* respectively (Button, Rayfield & Barrett, 2014). The bite force of *Stegosaurus stenops* (USNM 4934) has been estimated at between 140 and 275 N depending on the bite position along the jaw, modelled as sufficient to bite through smaller braches and leaves (Reichel, 2010). Further investigation of individual taxa will contribute to a broader picture of cranial evolution within Dinosauria.

## Conclusion

The retrodeformation of three ornithomimosaurian skulls has allowed for greater insight into ornithomimosaur cranial anatomy and function than was possible with deformed skulls, particularly the reconstruction of the myology and rhamphothecae. The reconstructions and functional interpretations presented here should be treated as biologically informed hypotheses of musculoskeletal anatomy that can inform on future myological, endocranial and biomechanical studies.

## Supplemental Information

10.7717/peerj.1093/supp-1 Appendix S1 Ornithomimosaur cranial material observed by ARC for this study

10.7717/peerj.1093/supp-2 Supplemental Information 1 3D PDF of *Garudimimus* (A) *Garudimimus brevipes* GIN 100/18, original specimen. (B) *Garudimimus brevipes*, after retrodeformation.

10.7717/peerj.1093/supp-3 Video S1 *Garudimimus brevipes* (GIN100/13) S1. *Garudimimus brevipes* (GIN100/13) cranium before reconstruction.

10.7717/peerj.1093/supp-4 Supplemental Information 2 3D PDF of *Ornithomimus* (A) *Ornithomimus edmontonicus* RTMP 95.110.1, original specimen. (B) *Ornithomimus edmontonicus*, after retrodeformation

10.7717/peerj.1093/supp-5 Supplemental Information 3 3D PDF of *Struthiomimus* (A) *Struthiomimus altus* RTMP 90.26.1, original specimen. (B) *Struthiomimus altus*, after retrodeformation.

10.7717/peerj.1093/supp-6 Video S2 *Garudimimus brevipes* (GIN100/13) reconstructed S2. *Garudimimus brevipes* (GIN100/13) reconstruction after retrodeformation.

10.7717/peerj.1093/supp-7 Video S3 *Struthiomimus altus* (RTMP 1990.026.0001) S3. *Struthiomimus altus* (RTMP1990.026.0001) skull before retrodefromation.

10.7717/peerj.1093/supp-8 Video S4 *Struthiomimus altus* (RTMP1990.026.0001) skull after retrodefromation S4. *Struthiomimus altus* (RTMP1990.026.0001) skull after retrodefromation.

10.7717/peerj.1093/supp-9 Video S5 *Ornithomimus edmontonicus* (RTMP 1995.110.0001) before retrodeformation S5. *Ornithomimus edmontonicus* (RTMP 1995.110.0001) skull before retrodeformation.

10.7717/peerj.1093/supp-10 Video S6 *Ornithomimus edmontonicus* (RTMP 1995.110.0001) skull after retrodeformation S6. *Ornithomimus edmontonicus* (RTMP 1995.110.0001) skull after retrodeformation.

## Institution abbreviations

GIN: Mongolian Academy of Sciences, Ulan Bator, Mongolia
RTMP: Royal Tyrrell Museum of Palaeontology, Drumheller, Alberta, Canada

## Myological abbreviations

AMEm: adductor mandibulae externus medialis
AMEp: adductor mandibulae externus profundus
AMEs: adductor mandibulae externus superficialis
AMP: adductor mandibulae posterior
PSTp: pseudotemporalis profundus
PSTs: pseudotemporalis superficialis
PTd: pterygoideus dorsalis
PTv: pterygoideus ventralis

## Additional Information and Declarations

### Data Deposition

The following information was supplied regarding the deposition of related data:
*Ornithomimus*

http://phenome10k.org/ornithomimus-edmontonicus-2/

 http://phenome10k.org/ornithomimus-edmontonicus/

*Struthiomimus*

http://phenome10k.org/struthiomimus-altus/

http://phenome10k.org/struthiomimus-altus-2/

*Garudimimus*

http://phenome10k.org/garudimimus-brevipes-2/

http://phenome10k.org/garudimimus-brevipes/

## References

[ref-1] Adams LA (1919). A memoir of the phylogeny of the jaw muscles in recent and fossil vertebrates. Annals of the New York Academy of Science.

[ref-2] Angielczyk KD, Sheets HD (2007). Investigation of simulated tectonic deformation in fossils using geometric morphometrics. Paleobiology.

[ref-3] Arbour VM, Currie PJ (2012). Analyzing taphonomic deformation of ankylosaur skulls using retrodeformation and finite element analysis. PLoS ONE.

[ref-4] Barsbold R (1981). Toothless carnivorous dinosaurs of Mongolia. Transactions, Joint Soviet-Mongolian Palaeontological Expedition.

[ref-5] Barsbold R, Perle A (1984). On first new find of a primitive ornithomimosaur from the Cretaceous of the MPR. Paleontologicheskii Zhurnal.

[ref-6] Bates KT, Falkingham PL (2012). Estimating maximum bite performance in Tyrannosaurus rex using multi-body dynamics. Biology Letters.

[ref-7] Bell PR, Snively E, Shychoski L (2009). A comparison of the jaw mechanics in hadrosaurid and ceratopsid dinosaurs using finite element analysis. The Anatomical Record.

[ref-8] Brochu CA (2000). A digitally-rendered endocast for *Tyrannosaurus rex*. Journal of Vertebrate Paleontology.

[ref-9] Button DJ, Rayfield EJ, Barrett PM (2014). Cranial biomechanics underpins high sauropod diversity in resource-poor environments. Proceedings of the Royal Society B.

[ref-10] Cooper RA (1990). Interpretation of tectonically deformed fossils. New Zealand Journal of Geology and Geophysics.

[ref-11] Curtis N, Kupczik K, O’Higgins P, Moazen M, Fagan MJ (2008). Predicting skull loading: applying multibody dynamics analysis to a macaque skull. Anatomical Record.

[ref-12] Dal Sasso C, Maganuco S, Buffetaut E, Mendez MA (2005). New information on the skull of the enigmatic theropod *Spinosaurus*, with remarks on its sizes and affinities. Journal of Vertebrate Paleontology.

[ref-13] Davies SJJF, Hutchins M, Jackson A, Bock WJ, Olendorf D (2003). Struthioniformes (Tinamous and Ratites). Grzimek’s animal life encyclopedia. 8 birds I tinamous and ratites to hoatzin.

[ref-14] De Klerk WJ, Forster CA, Sampson SD, Chinsamy A, Ross CF (2000). A new coelurosaurian dinosaur from the Early Cretaceous of South Africa. Journal of Vertebrate Paleontology.

[ref-15] Fairman JE (1999). Prosauropod and iguanid jaw musculature: a study on the evolution of form and function. Unpublished M.A. thesis.

[ref-16] Gunz P, Mitteroecker P, Neubauer S, Weber GW, Bookstein FL (2009). Principles for the virtual reconstruction of hominin crania. Journal of Human Evolution.

[ref-17] Haas G (1955). The jaw musculature in *Protoceratops* and in other ceratopsians. American Museum Novitates.

[ref-18] Haas G (1969). On the jaw muscles of ankylosaurs. American Museum Novitates.

[ref-19] Hedrick BP, Dodson P (2013). Lujiatun psitacosaurids: understanding individual and taphonomic variation using 3D geometric morphometrics. PLoS ONE.

[ref-20] Hieronymus TL, Witmer LM (2010). Homology and evolution of avian compound rhamphothecae. The Auk.

[ref-21] Holliday CM (2009). New insights into the dinosaur jaw muscle anatomy. The Anatomical Record.

[ref-22] Holliday CM, Witmer LM (2007). Archosaur adductor chamber evolution: Integration of musculoskeletal and topological criteria in jaw muscle homology. Journal of Morphology.

[ref-23] Hughes NC, Jell PA (1992). A statistical/computer-graphic technique for assessing variation in tectonically deformed fossils and its application to Cambrian trilobites from Kashmir. Lethaia.

[ref-24] Ji Q, Norell M, Makovicky PJ, Gao K, Ji S, Yuan C (2003). An early ostrich dinosaur and implications for ornithomimosaur phylogeny. American Museum Novitates.

[ref-25] Kobayashi Y (2004). Asian ornithomimosaurs. PhD Thesis.

[ref-26] Kobayashi Y, Barsbold R, Carpenter K (2005). Anatomy of *Harpymimus okladnikovi* Barsbold and Perle 1984 (Dinosauria; Theropoda) of Mongolia. The carnivorous dinosaurs.

[ref-27] Kobayashi Y, Lü J-C (2003). A new ornithomimid dinosaur with gregarious habits from the Late Cretaceous of China. Acta Palaeontologica Polonica.

[ref-28] Lambe LM (1902). On Vertebrata of the mid-Cretaceous of the North-west Territory. 2. New genera and species from the Belly River Series (mid-Cretaceous). Contributions to Canadian Palaeontology.

[ref-29] Lautenschlager S (2013). Cranial myology and bite force performance of *Erlikosaurus andrewsi*: a novel approach for digital muscle reconstructions. Journal of Anatomy.

[ref-30] Lautenschlager S, Witmer LM, Altangerel P, Rayfield EJ (2013). Edentulism, beaks and biomechanical innovations in the evolution of theropod dinosaurs. Proceedings of the National Academy of Sciences of the United States of America.

[ref-31] Lee YN, Barsbold R, Currie PJ, Kobayashi Y, Lee HJ, Godefroit P, Escuillié FO, Chinzorig T (2014). Resolving the long-standing enigmas of a giant ornithomimosaur *Deinocheirus mirificus*. Nature.

[ref-32] Makovicky PJ, Kobayashi Y, Currie PJ, Weishampel DB, Dodson P, Osmólska H (2004). Ornithomimosauria. The dinosauria.

[ref-33] Makovicky PJ, Li D, Gao K-Q, Lewin M, Erickson GM, Norell MA (2010). A giant ornithomimosaur from the Early Cretaceous of China. Proceedings of the Royal Society B.

[ref-34] Molnar JL, Pierce SE, Clack JA, Hutchinson JR (2012). Idealized landmark-based geometric reconstructions of poorly preserved fossil material: a case study of an early tetrapod vertebra. Palaeontologia Electronica.

[ref-35] Morhardt AC (2009). Dinosaur smoles: do the texture and morphology of the premaxilla, maxilla, and dentary bones of sauropsids provide the osteological correlates for inferring extra-oral structures reliably in dinosaurs?. MSc Thesis.

[ref-36] Motani R (1997). New technique for retrodeforming tectonically deformed fossils, with an example for ichthyosaurian specimens. Lethaia.

[ref-37] Norell MA, Makovicky P, Currie PJ (2001). The beaks of ostrich dinosaurs. Nature.

[ref-38] Osmólska H, Roniewicz E, Barsbold R (1972). A new dinosaur, *Gallimimus bullatus* n. gen., n. sp. (Ornithomimidae) from the Upper Cretaceous of Mongolia. Palaeontologia Polonica.

[ref-39] Perez-Moreno BP, Sanz JL, Buscalioni AD, Moratella JJ, Ortega F, Raskin-Gutman D (1994). A unique multitoothed ornithomimosaur from the Lower Cretaceous of Spain. Nature.

[ref-40] Perle A (1981). A new segnosaurid from the Upper Cretaceous of Mongolia. Transactions, Joint Soviet-Mongolian Palaeontological Expedition.

[ref-41] Porro LB, Rayfield EJ, Clack JA (2015). Descriptive anatomy and three-dimensional reconstruction of the skull of the early tetrapod *Acanthostega gunnari* Jarvik, 1952. PLoS ONE.

[ref-42] Rayfield EJ (2004). Cranial mechanics and feeding in *Tyrannosaurus rex*. Proceedings of the Royal Society B.

[ref-43] Rayfield EJ, Milner AC, Xuan VB, Young PG (2007). Functional morphology of spinosaur ‘crocodile-mimic’ dinosaurs. Journal of Vertebrate Paleontology.

[ref-44] Rayfield EJ, Norman DB, Horner CC, Horner JR, Smith PM, Thomason JJ, Upchurch P (2001). Cranial design and function in a large theropod dinosaur. Nature.

[ref-45] Reichel M (2010). A model for the bite mechanics in the herbivorous dinosaur Stegosaurus (Ornithischia, Stegosauridae). Swiss Journal of Geosciences.

[ref-46] Rushton AWA, Smith M (1993). Retrodeformation of fossils-a simple technique. Palaeontology.

[ref-47] Sanders RK, Smith DK (2005). The endocranium of the theropod dinosaur *Ceratosaurus* studied with computed tomography. Acta Palaeontologica Polonica.

[ref-48] Seki Y, Bodde SG, Meyers MA (2010). Toucan and hornbill beaks: a comparative study. Acta Biomaterialia.

[ref-49] Sereno PC, Zhao X-T, Tan L (2010). A new psittacosaur from Inner Mongolia and the parrot-like structure and function of the psittacosaur skull. Proceedings of the Royal Society of London B.

[ref-50] Snively E, Russell AP (2007). Functional variation of neck muscles and their relation to feeding style in Tyrannosauridae and other large theropod dinosaurs. The Anatomical Record.

[ref-51] Sternberg CM (1933). A new *Ornithomimus* with complete abdominal cuirass. The Canadian Field Naturalist.

[ref-52] Tahara R, Larsson HCE (2011). Cranial pneumatic anatomy of *Ornithomimus edmontonicus* (Ornithomimidae: Theropoda). Journal of Vertebrate Paleontology.

[ref-53] Tallman M, Amenta N, Delson E, Frost SR, Deboshmita G, Klukkert ZS, Morrow A, Sawyer GJ (2014). Evaluation of a new method of fossil retrodeformation by algorithmic symmetrization: Crania of Papionins (Primates, Cercopithecidae) as a test case. PLoS ONE.

[ref-54] Thomason JJ (1991). Cranial strength in relation to estimated biting forces in some mammals. Canadian Journal of Zoology.

[ref-55] Tschopp E, Russo J, Dzemski G (2013). Retrodeformation as a test for the validity of phylogenetic characters: an example from diplodocid sauropod vertebrae. Palaeontologia Electronica.

[ref-56] Weijs WA, Hillen B (1985). Cross-sectional areas and estimated intrinsic strength of the human jaw muscles. Acta Morphologica Neerlando-Scandinavica.

[ref-57] Williams SH, Bruton DL, Harper DAT (1990). Computer-assisted graptolite studies. Microcomputers in palaeontology.

[ref-58] Witmer LM, Ridgely RC (2009). New insights into the brain, braincase, and ear region of tyrannosaurs (Dinosauria, Theropoda), with implications for sensory organization and behavior. The Anatomical Record.

[ref-59] Young MT, Rayfield EJ, Holliday CM, Witmer LM, Button DJ, Upchurch P, Barrett PM (2012). Cranial biomechanics of *Diplodocus* (Dinosauria, Sauropoda): testing hypotheses of feeding behaviour in an extinct megaherbivore. Naturwissenschaften.

[ref-60] Zanno LE, Gillette DD, Albright LB, Titus AL (2009). A new North American therizinosaurid and the role of herbivory in ‘predatory’ dinosaur evolution. Proceedings of the Royal Society B.

[ref-61] Zanno LE, Makovicky PJ (2011). Herbivorous ecomorphology and specialization patterns in theropod dinosaur evolution. Proceedings of the National Academy of Sciences of the United States of America.

[ref-62] Zanno LE, Makovicky PJ (2013). No evidence for directional evolution of body mass in herbivorous theropod dinosaurs. Proceedings of the Royal Society B: Biological Sciences.

[ref-63] Zollikofer CPE, Ponce de León MS, Lieberman DE, Guy F, Pilbeam D, Likius A, Mackaye HT, Vignaud P, Brunet M (2005). Virtual cranial reconstruction of *Sahelanthropus tchadensis*. Nature.

