# Supplementary material for: Retrodeformation and muscular reconstruction of ornithomimosaurian dinosaur crania
Source: PeerJ. 2015 Jul 9;3:e1093. doi: 10.7717/peerj.1093 (PMC4512775; doi:10.7717/peerj.1093)
Supplement: Appendix S1 [file peerj-03-1093-s001.docx]

Appendix 1. Ornithomimosaur cranial material observed by ARC for this study.

| Museum | Number | Species |
| --- | --- | --- |
| Royal Tyrrell | 1990.026.0001 | *S. altus* |
| Royal Tyrrell | 1995.110.0001 | *O. edmontonicus* |
| ROM | 1790 | *S. altus* |
| ROM | 851 | *O. edmontonicus* |
| ROM | 840 | *O. edmontonicus* |
| IVPP | 11797-1 | *Sinornithomimus* |
