# Supplementary figures and images for: Retrodeformation and muscular reconstruction of ornithomimosaurian dinosaur crania
Source: PeerJ. 2015 Jul 9;3:e1093. doi: 10.7717/peerj.1093 (PMC4512775; doi:10.7717/peerj.1093)

*Garudimimus brevipes* GIN 100/18, original specimen

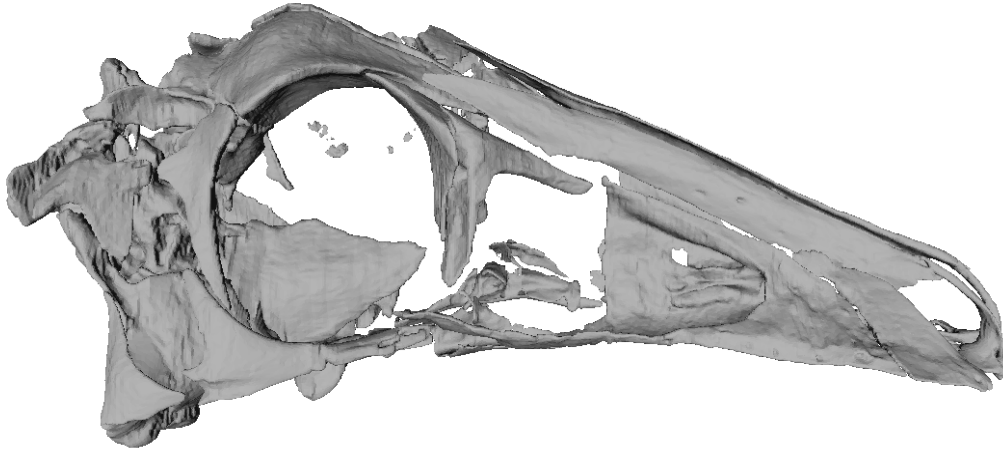

*Garudimimus brevipes*, after retrodeformation

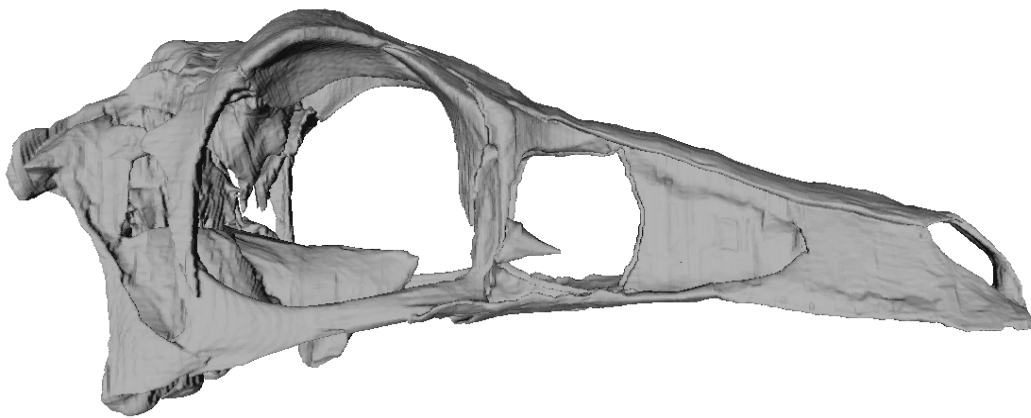

Supplement: Supplemental Information 1 — (A) Garudimimus brevipes GIN 100/18, original specimen. (B) Garudimimus brevipes, after retrodeformation. [file peerj-03-1093-s002.pdf]

*Struthiomimus altus* RTMP 90.26.1, original specimen

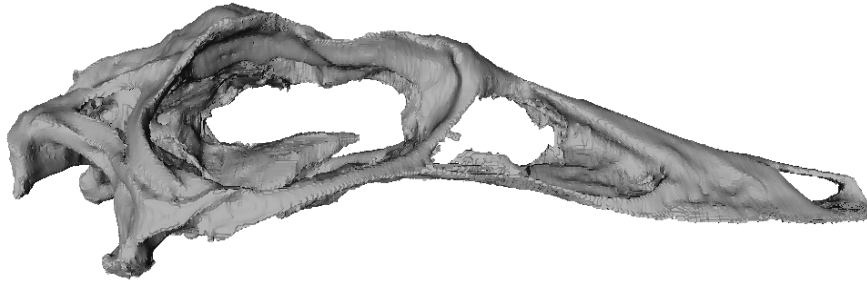

*Struthiomimus altus*, after retrodeformation

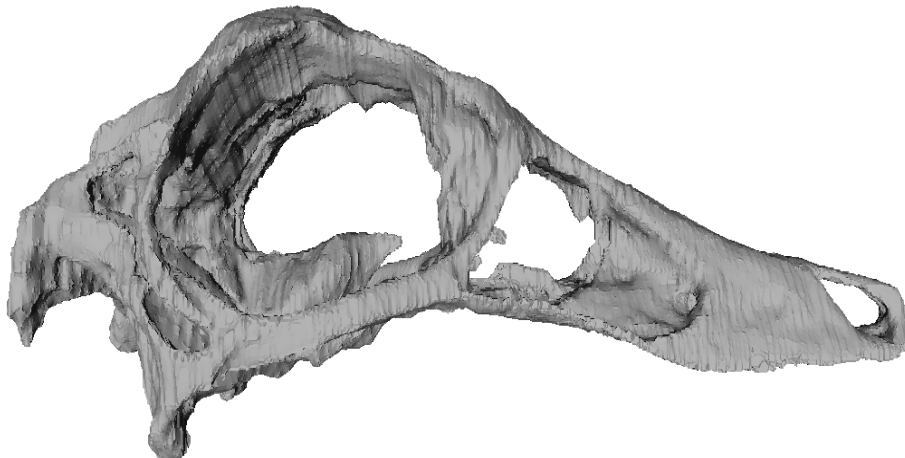

Supplement: Supplemental Information 3 — (A) Struthiomimus altus RTMP 90.26.1, original specimen. (B) Struthiomimus altus, after retrodeformation. [file peerj-03-1093-s005.pdf]
