# Supplementary material for: Retrodeformation and muscular reconstruction of ornithomimosaurian dinosaur crania
Source: PeerJ. 2015 Jul 9;3:e1093. doi: 10.7717/peerj.1093 (PMC4512775; doi:10.7717/peerj.1093)

Ornithomimus edmontonicus RTMP 95.110.1, original specimen

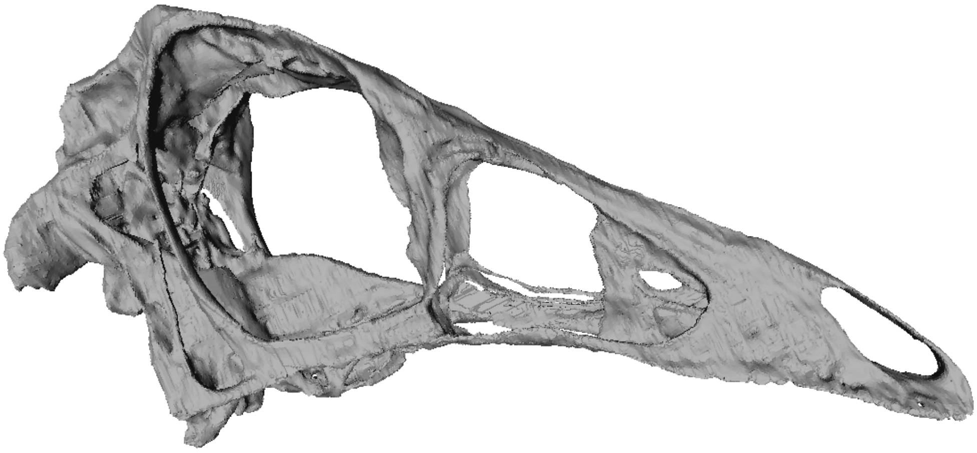

Ornithomimus edmontonicus, after retrodeformation

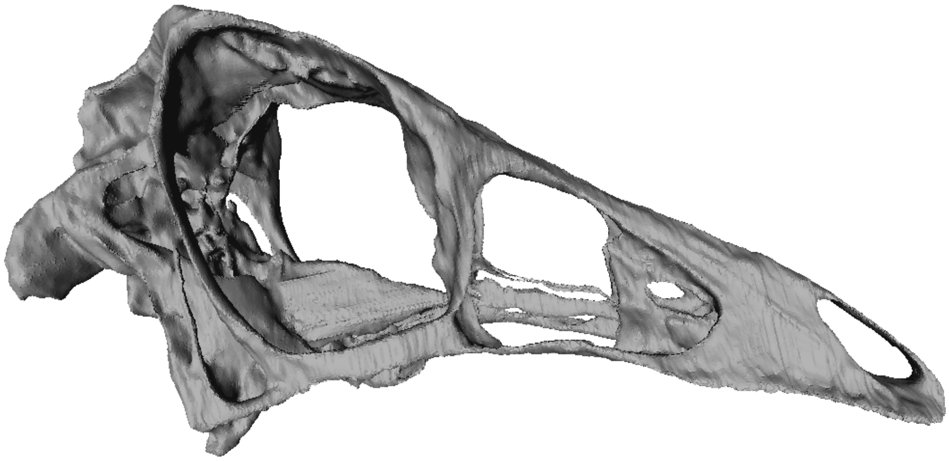

Supplement: Supplemental Information 2 — (A) Ornithomimus edmontonicus RTMP 95.110.1, original specimen. (B) Ornithomimus edmontonicus, after retrodeformation [file peerj-03-1093-s004.pdf]
